# Type 2 Inflammatory Responses in Autoimmune Demyelination of the Central Nervous System: Recent Advances

**DOI:** 10.1155/2019/4204512

**Published:** 2019-05-08

**Authors:** Massimo Costanza

**Affiliations:** Department of Clinical Neuroscience, Fondazione IRCCS Istituto Neurologico Carlo Besta, 20133 Milan, Italy

## Abstract

Type 2 immunity has long been confined to a restricted spectrum of responses, mostly including allergic reactions to innocuous environmental triggers. However, growing evidence suggests that cells and mediators typically associated with type 2 inflammation are involved in several physiopathological conditions, such as defense against toxic substances, anticancer immunity, and autoimmune diseases. In neuromyelitis optica, an autoimmune demyelinating disorder of the spinal cord and optic nerve, eosinophils extensively infiltrate lesions in the central nervous system (CNS) and promote tissue pathology in experimental models of this disease. Next-generation sequencing of CD4^+^ T cells isolated from a specific subtype of multiple sclerosis plaque has uncovered an unexpectedly Th2 profile of these cells. Even mast cells and other allergic mediators have been implicated in the modulation and/or effector mechanisms of autoimmune reactions against the CNS. In this review article, the most recent developments showing the involvement of type 2 inflammatory components in CNS autoimmunity are summarised and possible lines of further investigation are discussed.

## 1. Introduction

Type 2 immunity has an established role in counteracting macroparasite infections and in allergic inflammation, which might be viewed either as a misdirected immune reaction to harmless environmental triggers or, alternatively, as a protective response against noninfectious environmental toxins that leads to tissue pathology only when excessive [1]. CD4^+^ T helper (h)2 cells are crucial drivers of type 2 inflammation and secrete cytokines such as interleukin- (IL-) 13, IL-5, and IL-4, which induce antibody isotype class-switching in B lymphocytes to IgE and IgG1 secretion [1, 2]. Type 2 immune reactions also include components of innate immunity, such as eosinophils, mast cells (MCs) and basophils, and several mediators such as histamine [1, 2]. Conversely, type 1 immunity is mediated by Th1 and Th17 cells [1]. Th1 lymphocytes mainly release interferon- (IFN-) *γ* and tumor necrosis factor (TNF) and are generally involved in host defense against intracellular pathogens, by activating macrophage effector functions [3]. Th17 cells produce the cytokines IL-17A, IL-17F, IL-22, and granulocyte-macrophage colony-stimulating factor (GM-CSF) and orchestrate immune protection against certain extracellular pathogens, by recruiting neutrophils at the site of infection [4].

Recent developments have shown that allergic responses are involved in a plethora of immune functions, including venom detoxification, protection from noxious xenobiotics, and anticancer immunity [1, 5, 6]. Furthermore, numerous studies indicate that type 2 immune cells and mediators might exert important immunomodulatory and effector functions in autoimmune responses against the central nervous system (CNS), which are classically considered Th1- and Th17-mediated disorders [7]. In this review article, evidence indicating the involvement of allergic inflammation in neuromyelitis optica (NMO), multiple sclerosis (MS), and experimental models of CNS autoimmunity is presented and possible lines for future research are drawn.

## 2. Neuromyelitis Optica

Neuromyelitis optica, also known as Devic's disease, is a relapsing, inflammatory, and demyelinating disorder that primarily affects the optic nerve and the spinal cord [7]. The immunological hallmark of NMO, as well as its typical biomarker, is the presence of IgG targeting the astrocytic aquaporin (AQP)4 water channel in the serum of patients [8]. Pathological studies of active NMO lesions have depicted a uniform histological pattern among different patients, characterized by extensive demyelination in both grey and white matter, perivascular deposition of Ig and complement, and the infiltration of both T lymphocytes in small numbers and numerous macrophages/microglial-like cells [9].

One of the most peculiar features of the NMO plaque is the conspicuous presence of granulocytes and eosinophils with both an intact and degranulated morphology [9]. In an *in vitro* assay with cells expressing AQP4, the activation of eosinophils with NMO autoantibodies resulted in both antibody- and complement-dependent cell-mediated cytotoxicity [10]. Eosinophils have been also shown to increase tissue pathology in NMO lesions reproduced on spinal cord slices [10]. Interestingly, in a mouse model of NMO elicited by continuous intracerebral infusion of NMO-IgG and complement, tissue damage was enhanced in transgenic hypereosinophilic mice and reduced in mice depleted of eosinophils by an anti-IL-5 antibody compared to control mice. Moreover, the administration of cetirizine, a pharmacological agent with antihistamine and eosinophil-stabilizing properties, attenuated disease severity in this model [10].

The analysis of the cytokine and chemokine profile in the cerebrospinal fluid (CSF) of NMO patients has revealed significantly higher amounts of both Th17- and Th2-related cytokines, such as IL-6 and IL-13, with a similar tendency also for IL-5, even if it did not reach statistical significance [11]. T cells from NMO subjects exhibit greater proliferation to AQP4 and to its immune-dominant epitope p61-80, which shares 90% of sequence homology with a *Clostridium perfringens*-derived peptide, thus suggesting molecular mimicry as a potential pathogenic mechanism in this disease [12]. The analysis of IFN-*γ*- and IL-17-producing cells revealed that T lymphocytes specific for AQP4 p61-80, but not for the whole protein, display a Th17 polarization in NMO patients compared to healthy controls [12]. However, the percentages of CD4^+^ T cells specific for AQP4 whole protein or peptides producing Th2 cytokines were not evaluated in this work.

Dimethyl fumarate (DMF) has shown therapeutic efficacy in both MS and psoriasis, which are considered Th1/Th17- and Th17-mediated diseases, respectively [13]. One of the main mechanisms proposed for DMF efficacy in these disorders is the promotion of IL-4-producing Th2 cells, through the induction of type II dendritic cells [13]. In line with this hypothesis, the immune-phenotyping of peripheral blood cells from MS patients has confirmed that DMF treatment favors CD4^+^ T cell polarization toward a Th2 profile and results in the reduction of Th1/Th17 cells [14]. Notably, when DMF was administered in NMO patients, it triggered devastating relapses [15]. Therefore, a therapeutic strategy promoting a shift from Th1/Th17 to Th2 responses has been indicated as potentially deleterious in NMO [16].

## 3. Multiple Sclerosis

Multiple sclerosis is a chronic inflammatory disorder of the CNS affecting about 2.5 million people worldwide [17]. In most of the patients, the disease starts with recurrent episodes of reversible neurologic disability (relapsing-remitting MS (RRMS)) and later evolves to relentless progression of neurologic dysfunction (secondary-progressive MS) [18, 19]. A minority of patients experience a progressive course of the disease since the initial stages (primary progressive MS (PPMS)) [18]. The aetiology and pathogenic mechanisms in MS are still incompletely understood. It has been hypothesized that a detrimental interaction between genetic and environmental factors generates a T cell-driven autoimmune response against myelin in the CNS, resulting in the formation of multifocal areas of inflammation, extensive demyelination, and neurodegeneration [7, 20]. MS lesions are located in several areas of the CNS as periventricular white matter, optic nerves, corpus callosum, cerebellum, subpial cortex, and spinal cord [20]. Based on their histopathological features, active white matter lesions have been classified into three different types [18]. The most frequent lesion types (patterns I and II) are characterized by an important infiltration of mononuclear phagocytes and T cells [18]. Additionally, pattern II plaques display the deposition of Ig and an activated complement [18]. Pattern III lesions exhibit oligodendrocyte apoptosis, accompanied mainly by macrophage infiltration at plaque borders [18]. In each patient, there is preferentially a single kind of lesion, suggesting a main effector mechanism promoting disease progression in each individual [18].

Immune phenotyping of leukocytes in the peripheral blood of MS patients has uncovered a higher frequency of CD4^+^ T cells with high avidity for myelin peptides in comparison with healthy subjects and that these cells are significantly skewed toward a Th1-polarized profile [21, 22]. A later study has shown that in clinically active MS, a selective expansion of myelin-specific Th17 cells rather than Th1 occurs [23].

Recent work has characterized T cell clones infiltrating pattern II lesions derived from brain bioptic tissue of a SPMS patient [24, 25]. These plaques were characterized by Ig and complement deposition and the infiltration by plasma cells and mononuclear cells. By performing TCR next-generation sequencing, this study succeeded to identify T cell clones expanded in these lesions, isolate them from the cerebrospinal fluid (CSF), and functionally characterize these clones [24]. Surprisingly, authors found that CD4^+^ T cells with a Th2 signature accumulate in these pattern II plaques. CSF-infiltrating T cells from this patient secreted preferentially IL-4, IL-5, and IL-13 following *in vitro* restimulation. Moreover, one of these clones was shown to provide help to B cells for antibody production *in vitro*. This Th2 shift was restricted to T cells isolated from the CSF, as T lymphocytes purified from the peripheral blood of the same patient mainly released Th1 cytokines [24]. These results indicate a possible discrepancy in the immunological features of T cells isolated from the peripheral compartment compared to lymphocytes isolated from the CNS. Conversely, T cells infiltrating pattern III lesions of another SPMS patient displayed a Th1 profile, suggesting that the polarization toward a Th2 profile is specific for pattern II plaques [24]. Interestingly, gene microarray analysis of plaques derived from three out of four SPMS patients has detected significantly increased levels of the eosinophil cationic protein, a protein released upon eosinophil degranulation [26].

The opticospinal variant of MS, mostly affecting the Asiatic population, shares several clinicopathological features with NMO [27]. Notably, cytokine profiling of CSF samples from these patients has measured increased concentrations of IL-1*β*, IL-17, and IL-13 compared to controls and higher amounts of IL-17 and IL-5 compared to “classical” MS [27]. Furthermore, the analysis of CD4^+^ T cells has shown significantly enhanced percentages of IFN-*γ*^−^ IL-4^+^ T cells in the CSF of opticospinal MS in comparison to classical MS [27].

## 4. Pathogenicity of CD4^+^ T Cells Reactive against Myelin Antigens

The concept that Th1 and Th17 cells might represent the major drivers of the autoimmune attack in CNS autoimmune responses has been indirectly corroborated by data obtained in experimental autoimmune encephalomyelitis (EAE), an inflammatory demyelinating disease of the CNS, widely used as an animal model for MS [7]. EAE can be induced in several species, including primates and rodents, by immunization with either myelin proteins or immunodominant myelin peptides supplemented with adjuvants (active EAE) or by the adoptive transfer of myelin-specific T cells (passive EAE). Two extensively used models of active EAE include the chronic- (C-) EAE, obtained in C57BL/6 mice by immunization with myelin oligodendrocyte glycoprotein peptide 35–55 (MOG_35–55_), and the relapsing-remitting- (RR-) EAE, elicited in SJL/J female mice by immunization with proteolipid protein peptide 139–151 (PLP_139–151_) [28]. Both C-EAE and RR-EAE are considered to be mediated by Th1/Th17 myelin-reactive CD4^+^ T cells [7]. Interestingly, the injection of myelin-specific T lymphocytes polarized *in vitro* toward Th1 or Th17 profiles into SJL/J mice results in two different types of CNS autoimmune demyelination, which are indistinguishable from a clinical point of view but significantly different in terms of the inflammatory composition of CNS lesions [29]. Indeed, while Th1-induced EAE is dominated by the accumulation of macrophages, Th17-mediated EAE is characterized by an extensive infiltration of neutrophils within the CNS [29].

However, not only Th1 and Th17 cells can promote autoimmune neuroinflammation. Indeed, the injection of myelin-specific Th2 cells into immune-deficient RAG-1 KO mice provokes demyelination of the CNS, accompanied by a robust infiltration of granulocytes and mast cells [30]. A later study has shown that myelin-specific Th2 cells producing high levels of IL-5 can trigger a fatal ascending paralysis in RAG-1 KO mice [31]. Furthermore, in a marmoset model of EAE, a tolerization procedure with MOG results in early protection from acute disease but the late onset of a lethal demyelinating disorder, associated with a Th2 shift of myelin-reactive T cells and increased titres of MOG-reactive autoantibodies [32]. Overall, these data have provided a proof of concept that, in specific settings, Th2 cells can also be harmful in CNS autoimmunity.

## 5. Mast Cells in CNS Autoimmunity: The Enigma Might Still Be Unsolved

Mast cells are the key effector cells of allergic and anaphylactic reactions, when, after sensitization and reexposition to the allergen, they undergo IgE-mediated degranulation and massively release a plethora of preformed mediators, such as histamine, TNF-*α*, IL-13, and leukotrienes [33]. Neuropathological studies have detected MCs in the MS brain as early as in 1890 [34]. In recent years, MCs and MC-related transcripts, such as tryptase and *β* chain of the high-affinity receptor for IgE (Fc*ε*RI*β*), have been found increased in chronic MS plaques [26, 35, 36]. However, the large number of studies investigating the contribution of MCs in experimental models of CNS autoimmune pathology has provided puzzling results, which have not permitted to draw a uniform view on the role of these cells in EAE and MS [37–39].

The analysis of the biological functions of MCs has relied for many years on *Kit* mutant mice, such as the WBB6F_1_-*Kit*^W/W-v^ and C57BL/6-*Kit*^W-sh/W-sh^ strains [40]. These mice harbour a different kind of mutations affecting the expression of the tyrosine kinase receptor c-*Kit* and exhibit several phenotypic abnormalities, including MC deficiency. In particular, *Kit*^W/W-v^ mice display defective melanogenesis, sterility, anemia, and neutropenia and lack interstitial cells of Cajal (ICCs), while *Kit*^W-sh/W-sh^ mice are deficient of ICCs and melanocytes but are affected by splenomegaly and higher numbers of neutrophils, platelets, and basophils [40]. Due to these other phenotypic defects, to prove that a different biological response between *Kit* mutant and wild type mice is specifically dependent on the lack of mast cells, a “MC-reconstitution” or “MC-knock-in” experiment is necessary. In other words, it is evaluated whether the engraftment of *in vitro* bone-marrow-derived MCs (BMMCs) in *Kit* mutant mice restores the wild-type phenotype. By this approach, MCs have been implicated in a wide array of physiopathological conditions, including T cell and antibody-dependent autoimmunity [41], tumor growth [42], and tolerance to skin allograft [43, 44].

In the context of CNS autoimmunity, MCs have been demonstrated as detrimental in the pathogenesis of chronic EAE elicited in *Kit*^W/W-v^ mice (WBB6F_1_ background) with a specific protocol of disease induction (i.e., two rounds of immunization with high doses of MOG_35-55_ and adjuvants) [45]. In these specific model and experimental conditions, MCs have been shown to act both as immunomodulatory cells, by supporting the proinflammatory potential of myelin-reactive T cells in lymphoid organs [46], and as effector cells, by facilitating the infiltration of neutrophils within the CNS through TNF secretion [47]. Of note, in this model, not only the intravenous but even the intracranial reconstitution of *Kit*^W/W-v^ mice is sufficient to recapitulate the wild-type disease course [47]. An independent study has confirmed that *Kit*^W/W-v^ mice immunized with the same “high-dose” protocol display reduced EAE severity compared to control mice [48]. However, in other experiments utilizing different strategies for disease induction (i.e., a single immunization with medium doses of MOG_35-55_ and adjuvants), *Kit*^W/W-v^ mice develop EAE with a similar or even slightly increased severity than wild-type mice [48–50]. In the *Kit*^W-sh/W-sh^ model on the C57BL/6 background, a trend has been observed toward a similar or slightly exacerbated disease course and increased proinflammatory Th1/Th17 profile of encephalitogenic T cells in *Kit* mutant mice compared to controls [48, 49, 51]. Mast cell reconstitution is effective in *Kit*^W-sh/W-sh^ mice with EAE only when MCs are injected during the induction phase of the disease [51] but not when they are transplanted before the immunization [48]. The reasons for this discrepancy have not been clarified. However, in the classical MC-knock-in experiments, MCs are transplanted before the induction of the biological response under investigation. Indeed, this approach is supposed to favor BMMC adaptation to the host microenvironment and the acquisition—at least histologically—of the phenotype of endogenous MCs [33]. Overall, data obtained with *Kit* mutant mice suggest that MCs might play a clear and significant detrimental role in EAE only when a high-dose protocol of disease induction is applied in the WBB6F1-*Kit*^W/W-v^ strain.

More recently, the MC-reconstitution approach has been questioned, because the number and tissue distribution of injected MCs might not be physiological [39] and *in vitro*-derived MCs could behave differently than endogenously developed cells [39]. Alternatively, MC responses in conditions of neutropenia or neutrophilia of *Kit*^W/W-v^ or *Kit*^W-sh/W-sh^ mice, respectively, might represent the adaptation of MCs to the altered immune compartment of these specific mutant strains, and therefore, they might not be physiological. For all these reasons, in the last years, mouse strains with *Kit*-independent MC deficiency have been generated by utilizing different genetic strategies taking advantage of promoters from mast cell-specific genes, such as *Cpa3* and *Mcpt5*, to obtain MC depletion (reviewed in [52]). These new mouse models seem to bear less phenotypic abnormalities than *Kit* mutant mice and have restrained the numerous functions previously ascribed to MCs [39].

The C57BL/6-*Cpa3*^Cre/+^ (“Cre-Master”) strain is deficient of both mucosal and connective-tissue MCs and has a partial reduction of splenic basophils, due to the genotoxic effect of sustained synthesis of Cre-recombinase in Cpa3-expressing cells [50]. This model has normal counts of immature and mature B cells, naïve, activated, and memory CD4^+^ and CD8^+^ T lymphocytes, dendritic cells, macrophages, and neutrophils [50]. When active EAE is induced in this strain by a single immunization with medium doses of MOG_35-55_ and adjuvants, no clinical difference between *Cpa3*^Cre/+^ and *Cpa3*^+/+^ mice is observed and comparable levels of IFN-*γ*-producing CD4^+^ T cells in response to MOG_35-55_ restimulation are detected in both groups [50]. Moreover, the authors found that *Kit*^W/W-v^ mice develop EAE with a disease course similar to *Cpa3*^+/+^ mice [50]. Based on these results, MCs have been proposed to play a redundant role in EAE and the previous detrimental effects described for these cells have been considered actually an artifact related to *Kit* mutations [37, 39, 50].

However, these data do not provide conclusive evidence to contest MC involvement in T cell-mediated autoimmunity of the CNS. Indeed, results obtained with *Cpa3*^Cre/+^ and *Kit*^W/W-v^ mice cannot be directly compared, because *Kit*^W/W-v^ mice are on a mixed background (WB/ReJ×C57BL/6), while *Cpa3*^Cre/+^ mice are backcrossed for twelve generations on the C57BL/6 background. In the work by Feyerabend and colleagues, the clinical course of EAE in *Kit*^+/+^ mice (the proper control group for *Kit*^W/W-v^ derived from the colony) is not shown [50]. It is unknown how the genetic diversity between these two backgrounds impacts on the clinical development and pathogenic mechanisms driving CNS autoimmunity in these models. Furthermore, the protocol for EAE induction in this work significantly differs from the one utilized by Secor et al. [45]. New data have proved that even in *Kit*-independent models of MC deficiency, MCs are “tunable” immune players, depending on the strength and type of the immune response, thus suggesting this as an intrinsic feature of MC biology. As an example, while MCs promote inflammation in a mild model of contact hypersensitivity (CHS) [53], they dampen inflammatory responses in a model of severe CHS by IL-10 secretion [54, 55]. Therefore, it is still possible that MCs play a detrimental role in CNS autoimmunity in a specific genetic background (e.g., WBB6) and experimental conditions (i.e., high doses of MOG and adjuvants and two rounds of immunization). In other words, the reversion of the *Kit*^W/W-v^ mutant phenotype to wild-type EAE after MC engraftment as described by Secor et al. might still rely on MCs, independently on the limitations of the “MC-reconstitution” approach and independently of other phenotypic abnormalities related to *Kit* mutations.

Studies performed with MC-deficient *Cpa3*^Cre/+^ mice took advantage of the chronic EAE model, elicited by a single immunization with MOG_35-55_ peptide of mice on the C57BL/6 background. This represents a specific model of EAE that recapitulates some features of MS while having limitations at the same time [28]. Rather than relegating MCs to a redundant immune player in T cell-mediated autoimmunity of the CNS, it might be worth to deeper verify whether and the reason why, under certain circumstances, MCs might importantly enhance neuroinflammation. Indeed, MS is a heterogeneous disease, in terms of clinical expression, histopathological patterns of lesions, and their relative distribution in the CNS. A recent report has detected MCs and T cells colocalizing at the meningeal interface of the MS brain during the acute stage of the disease [56]. However, while T cells were found in all MS samples analyzed, MCs were identified in four out of eleven cases, suggesting they might be implicated only in a subset of patients. Meningeal reconstitution of MCs in the *Kit*^W/W-v^ strain has demonstrated that the production of IL-1*β* and TNF by MCs at the meningeal interface is important for the optimal encephalitogenicity of T cell responses and for neutrophil infiltration of the CNS, respectively [47, 56]. Of note, recent studies have found increased neutrophil markers in the early/acute stage of MS and have argued for a role of neutrophils in the breach of the blood-brain barrier (BBB) and, consequently, in nascent MS and EAE lesions [57].

In EAE literature, one of the most evident cases of controversy is related to the role of IL-17 in the pathogenesis of the disease. Indeed, Komiyama et al. reported that IL-17-deficient mice exhibit a significantly milder disease than wild-type mice, suggesting a detrimental function for IL-17 in MOG_35-55_-induced chronic EAE [58]. A few years later, Haak and colleagues demonstrated that IL-17-deficient mice develop EAE with a severity not significantly different from controls [59] and has proposed IL-17 as a marginal cytokine in the pathogenesis of EAE. In the first study, highlighting the importance of IL-17 in MOG-induced EAE, the disease was obtained with a protocol almost identical to one used by Brown's group (i.e., high doses of MOG and adjuvants and two rounds of immunization); in the second work contesting the role of IL-17 in EAE, the disease was elicited with a protocol similar to the one utilized by Feyerabend and colleagues (a single immunization with medium doses of MOG and adjuvants). Mast cells activated *in vitro* with IgE and antigen have been shown to break regulatory T (Treg) cell anergy and suppression, while promoting Th17 cell differentiation through IL-6- and OX-40L-dependent mechanisms [60]. Moreover, the presence of activated MCs in cocultures of Treg cells and effector T (Teff) cells licenses Teff cells to secrete specifically IL-17 but not Th1 and Th2 cytokines, such as IFN-*γ* and IL-4 [60]. Supernatants of activated human MCs selectively promote the expansion of IL-17-producing T cells from the pool of human memory CD4^+^ T lymphocytes, by an IL-1*β*-dependent mechanism [61]. In line with these findings, activated MCs significantly induce *in vitro* the gene expression of IL-17 and GM-CSF in MOG-specific T cells isolated from EAE mice, through IL-1*β* secretion [56]. Based on these considerations, it might be interesting to evaluate EAE development in *Cpa3*^Cre/+^ mice immunized with a high-dose immunization protocol, similar to the one utilized by Secor et al. [45]. Alternatively, it might be relevant to understand whether MCs are implicated in models of T cell-mediated autoimmunity of the CNS where the IL-17 cytokine is unequivocally involved, such as RR-EAE. Indeed, while IL-17 depletion significantly hampers disease progression in RR-EAE [62], it has no effect in C-EAE [59]. Moreover, RR-EAE better recapitulates the most common clinical form of MS, RRMS, in comparison to MOG-induced EAE [28]. The group of Brown has already shown that SJL/J*-Kit*^W/W-v^ mice develop lessened EAE than controls, thus indicating that MCs might be pathogenic in this model [63]. However, a demonstration in a *Kit*-independent MC-deficient model could be valuable to corroborate these findings in RR-EAE and overcome the ambiguity related to *Kit* abnormalities.

## 6. Other Allergic Mediators Implicated in MS and EAE

Histamine (HA) is an important mediator in a broad spectrum of physiological activities, ranging from the regulation of vascular permeability to neurotransmission, from the control of gastric secretion to immune modulation [64]. Synthesized from histidine by a unique enzymatic reaction mediated by histidine decarboxylase (HDC), histamine activates four types of heptahelical G-protein-coupled membrane receptors (HR_1-4_) [64].

In the context of immune responses, HA is widely accepted as one of the chief effector molecules in Th2-driven allergic reactions, when it is massively released from intracellular granules stored into MCs and basophils [65]. However, HA is endowed with complex immunomodulatory properties. When directly incubated with polarized human T lymphocytes, HA promotes Th1 responses through H1R and downmodulates Th1 and Th2 responses through H2R [66]. Other work has shown that HA sustains a Th2 environment indirectly, through the stimulation of dendritic cells (DCs) and monocytes [67, 68].

Raised concentrations of HA have been measured in the CSF of MS patients, but not all studies have confirmed this finding [69]. Gene microarray analysis has detected increased *H1R* transcripts in chronic MS plaques [26]. Furthermore, the gene profile of HRs on PBMCs isolated from different types and stages of MS has revealed that *H1R* mRNA levels are significantly downmodulated in PBMCs isolated from SPMS patients compared with healthy controls, while *H4R* transcripts are augmented in this group in comparison to both healthy donors and RR-MS [70]. H1R and H2R have been detected on mononuclear cells infiltrating the brain of mice with RR-EAE [71].

Both genetic and pharmacological approaches have investigated the contribution of HA and its receptors to the pathogenesis of EAE. Mice deficient for H1R develop less severe MOG_35–55_-induced chronic EAE than wild-type mice [72], and treatment with H1R antagonists lessens clinical symptoms of both RR-EAE [71] and rat EAE [73]. Myelin-specific T cells isolated from H1R-KO mice with EAE exhibit reduced production of IFN-*γ* and enhanced IL-4 secretion [72, 74], indicating a detrimental effect of H1R in CNS autoimmune pathology. Nonetheless, H1R expressed specifically by endothelial cells seem actually to reduce BBB permeability and protect from CNS autoimmunity [75].

Discordant results have been obtained when analyzing H2R functions in EAE. Indeed, while chronic EAE severity is decreased in H2R-deficient mice [76], treatment with a specific H2R agonist also prevents the disease [77].

The expression of H3R is mostly confined to the peripheral and central nervous systems, where it regulates the release of HA and other neurotransmitters at the presynaptic level [78]. Mice with targeted deletion of H3R have no alterations in the peripheral immune response against myelin but develop exacerbated EAE in comparison to controls, due to a dysregulation of the BBB permeability [78].

Genetic and pharmacological approaches have shown that also H4R contributes to dampening CNS autoimmunity. Indeed, H4R-deficient mice develop a more severe chronic EAE, associated with an increased CNS infiltration by Th17 cells and reduced numbers of Treg cells [79]. In the same work, H4R has been demonstrated to support the suppressive capacity of Treg cells [79]. Similarly, H4R antagonism with the JNJ7777120 compound triggers the exacerbation of both clinical and pathological features of EAE and the expansion of IFN-*γ*-producing cells within the lymph nodes in comparison with vehicle-treated mice [80].

In *Hdc*^−/−^ mice, which are unable to produce HA, chronic EAE develops with greater clinical severity, associated with an augmented secretion of proinflammatory cytokines, such as IFN-*γ* and TNF-*α* compared to wild-type mice [81]. *Hdc*^−/−^ mice display also a peculiar infiltration of polymorphonuclear cells and eosinophils within the CNS [81]. Surprisingly, Saligrama and colleagues have induced chronic EAE in mice lacking all the four HRs known so far, but both the clinical expression of the disease and Th1/Th17 autoreactive responses in these mice are significantly attenuated if compared to controls, thus suggesting the existence of a still unknown receptor for HA that might promote CNS autoimmune inflammation [82].

Recently, an antibody targeting the *α* chain of the high-affinity receptor for IgE (Fc*ε*RI) has been tested in chronic EAE [83]. The treatment with this antibody (MAR-1) promotes the complete depletion of basophils in the blood, lymph nodes, and spleen, without affecting the percentages of monocytes and T and B lymphocytes. MAR-1-treated mice develop exacerbated EAE and enhanced Th1 and Th17 responses against myelin antigen if compared to an isotype control-injected group. Based on these results, it has been postulated that basophils might be important sources of mediators that counteract Th1/Th17 responses against myelin in this model [83].

## 7. Concluding Remarks

**Figure 1:**
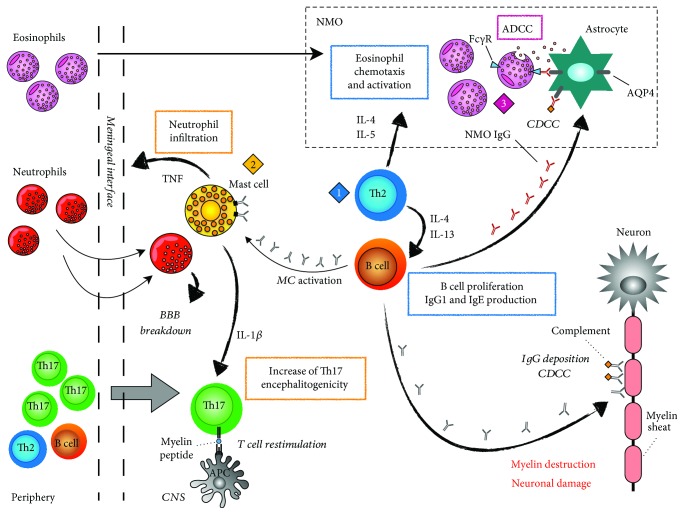
Potential mechanisms of CNS autoimmune inflammation driven by type 2 immune cells and mediators. (1) Passive transfer of myelin-specific Th2 cells can trigger inflammation and demyelination in experimental models of central nervous system (CNS) autoimmunity [30, 31]. Of note, autoreactive Th2 cells have been found in pattern II plaques of secondary-progressive (SP)MS [24, 25]. Furthermore, increased concentrations of IL-13 have been detected in the cerebrospinal fluid (CSF) of NMO [11] and the opticospinal variant of MS, which shares several features with NMO [27]. Higher percentages of IFN-*γ*^−^ IL-4^+^ T cells have also been detected in the CSF of opticospinal MS compared to classical MS [27]. Th2 cells might amplify autoimmune responses through the secretion of IL-13 and IL-4, key cytokines for the induction of B cell proliferation and Ig isotype switch toward IgG1 and IgE production [1, 2]. IgG and complement deposition are key features of both pattern II MS and NMO lesions [9, 18], suggestive of complement-dependent cell cytotoxicity (CDCC) processes. Moreover, IgE without antigen binding can induce the activation and the release of inflammatory cytokines by mast cells (MCs) [33]. (2) Mast cells are CNS-resident immune cells and have been localized at the meningeal interface in both EAE and in a subset of MS patients [47, 56]. MCs have been proposed to sustain CNS autoimmunity through at least two mechanisms: (i) MC-derived TNF supports CNS infiltration of neutrophils [47], which have been suggested as first-line amplifiers of EAE and MS inflammatory lesions [57], by promoting blood-brain barrier (BBB) breach [47]; (ii) MC-release of IL-1*β* can enhance the encephalitogenic potential of CNS-infiltrating myelin-reactive Th17 cells that are restimulated by local antigen presenting cells (APC) [56]. (3) Th2 cytokines IL-4 and IL-5 are two crucial chemoattractants and growth factors of eosinophils, which infiltrate NMO lesions [9]. Eosinophils can bind aquaporin 4- (AQP4-) specific IgG through the Fc*γ* receptor (Fc*γ*R) and mediate antibody-dependent cell cytotoxicity (ADCC) of AQP4-expressing astrocytes [10].

Studies discussed in this review article highlight that type 2 immunity might be implicated in autoimmune responses against the CNS (Figure 1). Immune cells and mediators typical of allergic inflammation have been found in NMO and, in certain circumstances, even MS, as suggested by the presence of Th2 cells in pattern II lesions of SPMS and by the colocalization of MCs and T cells in the meninges of a subset of patients. A common feature between NMO and pattern II MS lesions is the deposition of Ig and the complement, which suggests the involvement of humoral responses in the pathogenic processes driving tissue destruction in these disorders. Interestingly, in systemic lupus erythematosus (SLE), long considered a Th1- and Th17-driven disease with a strong autoantibody response, a Th2 environment and basophils have been recently shown to importantly promote disease development [84]. Deeper investigation is necessary to better understand the involvement of allergic components and their possible interplay with humoral responses in the context of CNS autoimmunity. Analyses in a larger cohort of MS patients are required to understand whether Th2 cells commonly infiltrate CNS lesions characterized by Ig deposition and to verify whether this Th2 shift is age-dependent. Indeed, brain aging has been recently shown to promote Th2 polarization of CNS-specific T cells at the choroid plexus [85].

In experimental models of CNS autoimmunity, it has been clearly demonstrated that the polarization of myelin-reactive CD4^+^ T cells has a significant impact on the outcome of the therapeutic regimen applied. Indeed, while neutralizing antibodies against IL-17 and GM-CSF protect from Th17-induced passive EAE in SJL/J mice, they are completely ineffective in counteracting Th1-mediated EAE in the same strain [29]. Even more strikingly, treatment with IFN-*β* reduces disease symptoms in passive EAE induced by Th1 cells but exacerbates Th17-mediated passive EAE in C57BL/6 mice [86]. These findings have been paralleled by data on MS, showing that patients unresponsive to IFN-*β* therapy exhibit significantly higher serum levels of IL-17F and IFN-*β* at the pretreatment stage compared to responder patients [87]. In opticospinal variants of MS and in NMO, the treatment with either IFN-*β* or DMF leads to the exacerbation of clinical symptoms [88–90]. Of note, both IFN-*β* and DMF have been shown to promote the Th2 profile [13, 14, 91]. Studies performed in experimental models of CNS autoimmunity have provided clear evidence of the possibly concomitant development of autoimmune and allergic reactions against myelin antigens. Indeed, in EAE models mediated by Th1/Th17 responses, the reexposure to the immunization antigen can promote the development of the most severe manifestation of allergy, that is, anaphylaxis [71, 92]. Overall, these data suggest that the potential occurrence of type 2 immune responses in NMO and in certain subtypes and/or stages of MS should be taken into consideration when designing and evaluating novel immunomodulatory approaches for the treatment of these diseases.
